# Supplementary material for: Structural basis for Vipp1 membrane binding: from loose coats and carpets to ring and rod assemblies
Source: Nat Struct Mol Biol. 2024 Oct 8;32(3):555–70. doi: 10.1038/s41594-024-01399-z (PMC11919686; doi:10.1038/s41594-024-01399-z)
Supplement: Supplementary file 1 — Supplementary Notes 1–7. [file 41594_2024_1399_MOESM1_ESM.pdf]

nature structural & molecular biology

Supplementary information https://doi.org/10.1038/s41594-024-01399-z

Structural basis for Vipp1 membrane binding: from loose coats and carpets to ring and rod assemblies

In the format provided by the authors and unedited

Structural basis for Vipp1 membrane binding: From loose coats and carpets to ring and rod assemblies

Benedikt Junglas[1], David Kartte[1,2], Mirka Kutzner[3], Nadja Hellmann[3], Ilona Ritter[1], Dirk Schneider[3,4], Carsten Sachse[1,2,5*]

[1]Ernst-Ruska Centre for Microscopy and Spectroscopy with Electrons, ER-C-3/Structural Biology, Forschungszentrum Jülich, 52425 Jülich, Germany.

[2]Department of Biology, Heinrich Heine University, Universitätsstr. 1, 40225 Düsseldorf, Germany.

[3]Department of Chemistry, Biochemistry, Johannes Gutenberg University Mainz, Germany.

[4]Institute of Molecular Physiology, Johannes Gutenberg University Mainz, Germany.

[5]Lead contact

* Correspondence: c.sachse@fz-juelich.de (C.S.)

Supplementary Notes

Characteristics Vipp1 assemblies 3

Vipp1 tubes 4

Vipp1 rings 5

Vipp1 Δα0 6

Plasticity-restrained Vipp1 7

Vipp1 carpets 9

Vipp1 refolded 11

Characteristics Vipp1 assemblies

Characteristics of Vipp1 assemblies observed upon membrane reconstituion

Complexes with different diameters and rotational symmetries were identified. The stacked rings have a regularly tapered appearance with maximum diameters of individual rings of ~200 – 280 Å. The stacked rings consist of joined end-to-end rings, presumably resulting from stacking of different rings. The Type I tubes displayed a spike pattern at the outside of the tubes connected by close-to-parallel lattice lines. Most Type I tubes are made of straight and regular stretches of 100 – 200 nm in length with an outermost diameter of 300 Å, interrupted by irregular parts made of bulges, indentations, or kinks. In contrast, Type II tubes were more regular along their length having distinct apparent outer diameters of ~250 – 260 Å and displayed a crisscross pattern without any spikes. The carpet assemblies were the most heterogeneous structures. In some cases, they completely covered vesicles and formed ordered almost tape-like tracks on the vesicle surface, while in other cases vesicles were only partially covered by discontinuous patches with spikes at the edges, closely resembling Type I tubes.

Vipp1 tubes

Detailed description of helical Vipp1 tubes

In Type I tubes of 305 Å diameter with a helical rise and rotation of 1.79 Å and 124.4º, respectively, the monomers with the characteristic α1-α2 hairpin are arranged almost parallel to the tube axis (**Fig. 2B, Ext. Data Fig. 2A+B**). In addition, we found Type II tubes with two different diameters. The smaller tubes had a diameter of 275 Å with a helical rise and rotation of 2.10 Å and 43.5º, respectively. The larger tubes had a diameter of 290 Å with a helical rise and rotation of 1.96 Å and 133.5º, respectively. Apart from the different diameters and helical symmetries, the structures of both Type II tubes were very similar to one another in that the monomers were arranged approximately 45° to the tube axis. Type I and Type II tube cross-sections showed density for an engulfed lipid bilayer in their lumen, with close contact of the Vipp1 monomers to the outer leaflet of the bilayer. The radial density profiles of the helical tubes with enclosed membranes revealed that the phosphate-to-phosphate headgroup distance (from here on bilayer thickness) in all three tubes remained nearly identical at 35 Å (**Fig. 2C**). The inner leaflet radius changed from 57 Å (Type I tubes) to 41 Å (small Type II tubes). In the Type I tube map, we were able to identify α-helical density for α0−5 whereas α6 was not resolved. In the core of the tube wall, the ESCRT-III-typical fold of the α1−4 were easily placed in the density (**Fig. 2D**). In the periphery, the tip of the α1−3 hairpin and α5 contact sites create the distinctive structural motif when seen in the side view (**Fig. 2E**). Although the polymeric assemblies differ in their architecture, the intermolecular contacts that stabilize the assemblies are nearly identical in Type I and Type II tubes (α1−4 contacts in the core, α1−3 hairpin-to-α5 contacts in the periphery). These or similar intermolecular contacts are conserved in Vipp1 rings, PspA rods, and eukaryotic ESCRT-III proteins [2,3,19]. To accommodate similar contacts in different topologies, the monomers adapt with changes at the flexible hinge regions 1, 2, and 3 (**Ext. Data Fig. 2C**). Within the Vipp1 Type I and Type II tubes, membrane contact is mediated by α0 that is lying flat in the membrane plane causing a local disturbance in the membrane, presumably by pulling the lipid headgroups towards the inner tube wall (**Fig. 2F, Ext. Data Fig. 2D**). Helix α0 appears to partially submerge into the outer leaflet of the bilayer.

Vipp1 rings

Detailed description of stacked Vipp1 rings

The individual rings in these assemblies had C11 to C14 rotational symmetry. Although we also found rings with higher/lower rotational symmetries, we were not able to generate reliable 3D reconstructions in those cases. Subsequently, we flexibly fitted models of the available Vipp1 ring structures[1,3] into our density maps, and found that the available models had good overlap with our reconstructions. Only minor adjustments at hinge 3 and α5 were necessary, presumably reflecting the poorer resolution and map quality of the stacked rings compared with the single ring structures (**Fig. 3A, Ext. Data Fig. 3A**). In those elongated structures, the rings are joined end to end resulting in a polar head-to-tail stacking: The more tapered side of one ring assembly (*i.e.,* the top side) is connected to the less tapered side (*i.e.,* the bottom side) of the next ring assembly. Unlike the individual rings, our stacked-ring assembly structures show bilayer density in the lumen with the inner membrane leaflet clearly discernable. As the outer leaflet is located close to the denser ring wall, it is not easily distinguished from the ring density. In analogy to the Type I and Type II helical tubes, α0 is lying flat on the membrane and appears to partially submerge into the outer leaflet (**Fig. 3B**). Due to the tapered nature of the rings, the membrane diameter changes over the z-axis of a ring. In the C12 rings, the inner leaflet diameter changes in accordance with the Vipp1 structures from 42 to 86 Å from the narrowest to the widest part of the ring (**Fig. 3C**). We observed similar effects for the other rings, and, as expected, the rings with the smallest diameters engulf the membranes with the smallest diameters (**Ext. Data Fig. 3B**). Interestingly, our maps include density of the adjacent upper and lower neighboring ring stacks. The contact between the rings is mediated by the interaction of the α1–3 hairpin of the lower ring to α5 of the upper ring. Additionally, the stacked rings include a continuous membrane tube engulfed by the stacked ring assembly. The engulfed membrane is most constricted at the position where two rings touch, further increasing the local curvature (**Fig. 3D, Ext. Data Fig. 3C**). In contrast to the thickness of the lipid bilayer engulfed by helical Vipp1 tubes, the bilayer thickness in stacked Vipp1 rings was generally smaller and more variable (**Ext. Data Fig. 3D**). First, the bilayer thickness within one ring varies from the narrowest to the widest part (*i.e.,* 32 to 34 Å in the C12 rings). Second, the bilayer thickness varied from the smaller to the larger rings (*i.e,.* 32 to 30 Å at the narrowest part of C11 to C14 rings).

Vipp1 Δα0

Helix α0 is critical for membrane tubulation and affects the Vipp1 polymer structure

In the absence of membranes, Vipp1(α1-6) formed large and straight rods of different diameters (**Ext. Data Fig. 4A**). In contrast to the full-length Vipp1, we did not observe the formation of any ring complexes nor any other polymers than Vipp1 rods, in agreement with previous observations[11]. After reconstitution with lipids, Vipp1(α1-6) formed again solely rods, similar to the rods observed in the absence of membranes. Additionally, in the micrographs we did not find any indications of Vipp1(α1-6) binding to membrane surfaces, neither protein carpets, nor loose coats on the membranes, nor deformed/irregularly shaped vesicles (**Ext. Data Fig. 4B**). The Vipp1(α1-6) rods had a large range of diameters from 280 to 420 Å according to the class averages and the radial density profiles of the helical reconstructions (**Ext. Data Fig. 4A-C**). We also compared the radial density profiles of Vipp1(α1-6) rods with full-length Vipp1 tubes in the presence of membranes (**Ext. Data Fig. 4D**). Strikingly, we could not find density for a tubulated membrane in the Vipp1(α1-6) rods. Therefore, we conclude that helix α0 is critical for membrane tubulation and engulfment by Vipp1 polymers. In total, we determined a series of ten unique rod structures with global resolutions of 6.3 to 7.8 Å, respectively, with the best-resolved parts in the center of the rod walls and poorer resolutions in the periphery (**Ext. Data Fig. 4E and F**). The Vipp1(α1-6) structures obtained in the absence and presence of lipids were mostly identical in the respective rods including the same helical symmetries. Again, we used PDB: 7O3W as the reference monomer structure and flexibly fitted it to the rod reconstructions. We noticed that the monomer arrangement was different from our full-length Vipp1 helical tubes and was more similar to *Syn*PspA rods (*i.e.,* the angle of the monomers was ~90 ° relative to the rod axis)[2]. However, the before-mentioned features of Vipp1 polymers remained conserved (α1–4 contacts in the core, α1–3 hairpin-to-α5 contacts in the periphery), and, as for the other Vipp1 assemblies, the monomers displayed a structural plasticity due to the conformational flexibility in the hinge regions 1-3 to adapt to the different rod diameters (**Ext. Data Fig. 5A and B**). In conclusion, α0 is critical for membrane interaction and tubulation of Vipp1 as well as in controlling the polymer architecture of Vipp1.

Plasticity-restrained Vipp1

Detailed description of plasticity-restrained Vipp1 assemblies

Mainly responsible for the associated conformational flexibility appears to be the loop region between α3 and α4 (Hinge 2) that enables different shapes and extensions of the Vipp1 monomers to adapt to different ring and rod diameters, *e.g.*, as demonstrated for the Vipp1(α1-6) rods (**Ext. Data Fig. 5B**) and Vipp1 rings[1,3]. Thus, we hypothesized that limiting the conformational flexibility of this region in the Vipp1 monomer may reduce the plasticity of Vipp1 assemblies observed during membrane interaction. For this purpose, we designed two Hinge 2 mutants (**Fig. 4A**). First, we created a mutant where the whole loop connecting α3 and α4 was removed (aa 157 – 166, Vipp1 dL10). Unfortunately, this mutant did not form any well-ordered assemblies suitable for more detailed structural characterization, suggesting that Hinge 2 is indeed critical for Vipp1 assembly formation (**Ext. Data Fig. 6A left**). Second, we replaced the loop region with a deca-alanine stretch (Vipp1 dL10Ala) to convert Hinge 2 to an α-helix, thus potentially stabilizing the conformation of large diameters while also limiting the structural flexibility of the loop. Interestingly, this mutant was capable of forming single-rings in the absence of membranes, although the rings had larger diameters and were not as uniformly shaped as the wildtype (WT) rings (**Ext. Data Fig. 6A right** and compare **Ext. Data Fig. 1A)**. Next, we analyzed the structure of Vipp1 dL10Ala in the presence of membranes using cryo-EM, as we did for the WT. We found that Vipp1 dL10Ala mostly formed Type II tubes (69%) including single-rings (27%), stacked rings (3%) as well as Type I tubes (2%) (**Fig. 4B, Ext. Data Fig. 6B**). In contrast to WT, we found only minor shares of two-dimensional carpet or stacked-ring structures and the observed tubular structures were straight in appearance without any kinks and bends but with a constant apparent diameter of 220 Å along the tube axis. Thus, the spectrum of assemblies found in the Vipp1 dL10Ala EPL sample was practically reduced to two types of assemblies (Type II tubes and single rings) making up approx. 95% of all assembly types. In contrast, in the WT EPL sample, all five assembly types were more or less evenly distributed. These data indicate that the replacement of Hinge 2 with 10 alanines indeed constrained the observed plasticity of Vipp1 assemblies.

Given the reduced plasticity of the Vipp1dL10Ala assemblies, we set out to determine the cryo-EM structures from this sample. First, we focused on single rings and were able to determine six structures ranging from C15 to C20 symmetry (**Ext. Data Fig. 6C)**. As observed in negative stain, the single rings were larger than the WT rings and showed C15 to C20 rotational symmetry with diameters from 330 to 435 Å, while the so far solved WT ring structures have C11 to C18 symmetry with approx. 240 to 350 Å diameters[1,3]. The resolution of our single ring reconstructions was limited to 7 to 10 Å resolution, presumably due to a limited homogeneity (**Ext. Data Fig. 6D**). Due to the small fraction of stacked rings and Type I tubes present in the Vipp1 dL10Ala sample, we were not able to generate reliable reconstructions for these assemblies. As for the WT, we identified two sizes of regular Type II tubes

with an engulfed lipid bilayer. The smaller tubes had a diameter of 250 Å with a helical rise and rotation of 2.53 Å and 62.4º, respectively. The larger tubes had a diameter of 270 Å with a helical rise and rotation of 2.13 Å and 53.15º, respectively. Apart from the different diameters and helical symmetries, the structures of both Type II tubes were similar to the WT Type II tubes (the monomers were arranged approximately 45° to the tube axis, and the engulfed membrane tubes had inner leaflet radii of 39 and 47 Å, respectively) (**Table 1, Fig. 4C+D**). The regular Type II tubes were resolved at 5.5 and 6.4 Å resolution, respectively (**Ext. Data Fig. 7A**). In addition to the regular Type II tubes, we found a subclass of Type II tubes making up approx. 46% of the overall sample, apart from 23 % regular Type II tubes. These Type IIb tubes formed left-handed instead of right-handed helices and also came in two sizes. The smaller tubes had a diameter of 260 Å with a helical rise and rotation of 6.54 Å and -157.0º in addition to C3 symmetry, respectively. The larger tubes had a diameter of 270 Å with a helical rise and rotation of 4.04 Å and -34.45º in addition to C2 symmetry, respectively. Apart from the different diameters and helical symmetries, the structures of both Type II tubes were similar to the WT Type II tubes with the monomers arranged approximately 45° to the tube axis. Both Type IIb tubes were resolved at 3.0 Å resolution, presumably due to the reduced flexibility of the Hinge 2 region (**Ext. Data Fig. 7A**), allowing model building at near-atomic resolution.

The role of α0 in stabilizing Vipp1 assemblies

As shown in this study (see **Ext. Data Fig. 4+5**), helix α0 was found to be critical for membrane tubulation while it also affects the Vipp1 polymer structure. Given its key position at the inner wall of the Vipp1 assembly, it contacts a total of four different subunits in Vipp1 assemblies by forming polar interactions via its own residues D5, R6, R9, R12, and E23 with subunit (a) α4 R174, subunit (b) α0 N16, E23 and α1 D35, subunit (d) α4 R170, and subunit (f) α0 R6 (**Fig. 4E**), which may explain the central importance of helix α0 for enabling different assembly states of Vipp1, *i.e.*, rings, Type I/II Tubes *vs*. solely rods in the Vipp1 (α1-6) sample. In the Type IIb tubes, α0 is not embedded in a continuous bilayer as in the regular Type II tubes. Instead, we found unassigned discontinuous density in the tube lumen, at a radius of 73 or 81 Å, respectively, at the similar radii where the head group peak of the bilayer of regular Type II tubes was found (see **Fig. 4C** highlighted in orange, **Fig. 4D** bottom overlay Type II and Type IIb). In line with previous observations[1], we propose that this density is caused by individual lipid molecules interacting with α0. The orientation of helix α0 with respect to the α1/α2 hairpin is identical in the Type IIb tubes in comparison with Type II and all previously described Vipp1 assemblies (single/stacked rings, Type I tubes (**Fig. 4E left** compare with **Ext. Data Fig. 2C** and **Ext. Data Fig. 7B-D**)). Moreover, as the radial position of α0 is identical at approx. 84 Å as well as helices α1/2 for bilayer-internalized Type II and membrane-free Type IIb assemblies, we will use the better resolved 3.0 Å structure of Type IIb assemblies for further side-chain based interpretations.

Vipp1 carpets

Analysis of Vipp1 assemblies on vesicles using cryo-electron tomography

As the membrane-attached Vipp1 assemblies were not suitable for 3D reconstruction from 2D images, due to limited views of preferred orientation, we elucidated the 3D structure of these assemblies by cryo-electron tomography (cryo-ET). Therefore, we collected a total of 123 tomograms from a Vipp1 lipid sample suitable for further analysis. Subsequent membrane segmentation using a progressively trained U-Net[30] revealed a large vesicle with several smaller encapsulated vesicles (**Fig. 5B** left, raw tomogram in **Ext. Data Fig 8C**) while the large vesicle is covered by Vipp1 assemblies, such as loose coats and larger carpets. Noteworthy, we also discovered tape-like spiraling ribbons of Vipp1 in our tomograms (**Fig. 5B** right, **Ext. Data Fig. 8D**). These highly curved 2D assemblies were only 1-2 nm thick and had lengths of several hundred nanometers. They were mostly found at the air-water interface or the carbon support film, indicating that they preferred to bind to distinct surfaces (*i.e.,* hydrophobic surfaces). Along their axis, they showed a very regular stripe pattern with 55 Å distancing, similar to the hairpin array distances found in Type I tubes and carpets (**Ext. Data Fig. 8E**). Vipp1 assemblies on larger vesicles (>200 nm) are mostly present in either small patches inducing bulges or tubes/rings creating membrane protrusions with positive membrane curvature (gallery of tomograms in **Fig. 5C**). Intermediate vesicles (around 100 nm) are almost fully covered with Vipp1 carpets, showing the characteristic spike pattern while they often have irregular shapes or large bulges. In some cases, we found intricate membrane networks with very high Vipp1 coverage and regions with high local membrane curvature. At last, we found small vesicles (15 – 80 nm) fully covered with Vipp1. Most small vesicles that were not encapsulated were covered with Vipp1 including the regular spike patterns. Notably, we could not clearly distinguish between very small Vipp1-membrane assemblies (<15 nm) and Vipp1 ring complexes with engulfed membranes in our tomograms.

For a more quantitative analysis of the observed Vipp1 membrane assemblies, we determined the membrane curvature for 123 tomograms (**Ext. Data Fig. 9A**). In addition, for each segmented membrane voxel region we checked for Vipp1 voxel presence and for each curvature we computed an occupancy share with zero representing empty and one fully covered membranes (**Fig. 6A** right, **Ext. Data Fig. 9B**). Based on the here determined values, membrane curvature correlates with Vipp1 occupancy (**Fig. 6A** left). The membrane occupancy increases linearly with increasing membrane curvedness between curvedness values of 0.02 to 0.06. For high curvature regions, *i.e.,* for curvednesses larger than 0.06, the graph indicated a saturation of Vipp1 membrane binding for high curvature regions. To support the assumption that Vipp1's affinity is higher for liposomes with high curvature, we compared binding of the protein to liposomes of different sizes (**Fig. 6B, Ext. Data Fig. 9C**). Employing

tryptophan fluorescence spectroscopy, we observed pronounced changes in the fluorescence emission spectra when the protein was incubated with increasing concentrations of sonified liposomes with an average diameter of ~55 nm as opposed to extruded liposomes with an average diameter of ~144 nm where hardly any change in tryptophan fluorescence was detected. This observed difference indicates that Vipp1 preferrably binds to small liposomes with high membrane curvature supporting the apparent Vipp1 coverage dependence on the curvature observed in the tomograms.

To further investigate the structure and organization of Vipp1 in the membrane-attached carpets, we excised subvolumes of 50 nm from the tomograms and performed subtomogram averaging of Vipp1-occupied membrane cubes, excluding small versicles (<15 nm) and tubes from the analysis. This way, we were able to determine three structures of Vipp1 carpets for curvedness 0.05, 0.054, and 0.058, at 20, 18, and, 19 Å resolution, respectively (**Table 2**, **Fig. 6C, Ext. Data Fig. 9D**). The reconstructed structures included density for the inner and outer membrane leaflet in addition to the Vipp1 lattice that was remarkably similar to Vipp1 ring complexes with respect to spike separation distance and monomer stacking distance. To generate an atomic model for our highest resolution structure (curvedness 0.054, 18 Å global resolution), we placed a lattice of a generated C20 monomer assembly (derived from PDB: 7O3Z) guided by the characteristic α1−3 hairpin-to-α5 spike shape in the periphery in the density (**Ext. Data Fig. 9E**). In accordance with previously described Vipp1 polymers, Vipp1 monomers form the typical α1−4 contacts in the core and α1−3 hairpin-to-α5 contacts in the periphery. Helix α0 presumably mediates membrane contacts. The observation that it was not possible to determine a structure for lower curvature Vipp1 carpets, despite abundant particle data, may indicate less membrane coverage and regularity in the arrangement of low curvature Vipp1 carpets, in agreement with recent AFM measurements on flat membrane surfaces[24].

Vipp1 refolded

Vipp1 is more reactive when refolded in the presence of membranes

Previous studies on Vipp1 membrane interaction worked with pre-formed ring complexes incubated with membranes[3,8,13,24,26].These studies suggested that preformed *Synechocystis* Vipp1 ring complexes in solution bind to membranes and afterward disassembled into carpet structures, while *Chlamydomonas* Vipp1 formed rod structures with internalized membranes. Clearly, the observations depend on the Vipp1 homolog and the exact preparation conditions used in the different studies. The observation that lipids are often copurified with pre-formed Vipp1 assemblies isolated from *E. coli* after heterologous expression could also lead to a decrease in apparent membrane binding and membrane remodeling[1,31]. Likely, pre-formed Vipp1 ring complexes are less reactive as they are already present in a polymeric form, as has been observed for the closely related *Syn*PspA[2]. Upon interaction with membranes, preformed Vipp1 polymers may just slowly disassemble due to slightly favoring protein-membrane interactions. Indeed, in a recent analysis of assembled *Synechocystis* Vipp1 ring complexes binding to a solid-supported bilayer, disassembly of the ring complexes on the membrane surface was observed via AFM[24,32]. Due to the solid support, the membrane may not be as flexible as a non-tethered membrane of liposomes, thus allowing the formation of carpets but not of membrane-attached rings or tubes.
