# Supplementary material for: Structural basis for Vipp1 membrane binding: from loose coats and carpets to ring and rod assemblies
Source: Nat Struct Mol Biol. 2024 Oct 8;32(3):555–70. doi: 10.1038/s41594-024-01399-z (PMC11919686; doi:10.1038/s41594-024-01399-z)
Supplement: Supplementary file 2 — Reporting Summary [file 41594_2024_1399_MOESM2_ESM.pdf]

nature portfolio

Corresponding author(s): Carsten Sachse

Last updated by author(s): Jul 4, 2024

Reporting Summary

Nature Portfolio wishes to improve the reproducibility of the work that we publish. This form provides structure for consistency and transparency in reporting. For further information on Nature Portfolio policies, see our Editorial Policies and the Editorial Policy Checklist.

Statistics

For all statistical analyses, confirm that the following items are present in the figure legend, table legend, main text, or Methods section.

n/a	Confirmed	
☐	☒	The exact sample size (*n*) for each experimental group/condition, given as a discrete number and unit of measurement
☐	☒	A statement on whether measurements were taken from distinct samples or whether the same sample was measured repeatedly
☒	☐	The statistical test(s) used AND whether they are one- or two-sided<br>*Only common tests should be described solely by name; describe more complex techniques in the Methods section.*
☒	☐	A description of all covariates tested
☒	☐	A description of any assumptions or corrections, such as tests of normality and adjustment for multiple comparisons
☐	☒	A full description of the statistical parameters including central tendency (e.g. means) or other basic estimates (e.g. regression coefficient) AND variation (e.g. standard deviation) or associated estimates of uncertainty (e.g. confidence intervals)
☒	☐	For null hypothesis testing, the test statistic (e.g. *F*, *t*, *r*) with confidence intervals, effect sizes, degrees of freedom and *P* value noted<br>*Give P values as exact values whenever suitable.*
☒	☐	For Bayesian analysis, information on the choice of priors and Markov chain Monte Carlo settings
☒	☐	For hierarchical and complex designs, identification of the appropriate level for tests and full reporting of outcomes
☒	☐	Estimates of effect sizes (e.g. Cohen's *d*, Pearson's *r*), indicating how they were calculated

Our web collection on statistics for biologists contains articles on many of the points above.

Software and code

Policy information about availability of computer code

Data collection	Digital Micrograph Version 3.32.2403.0 ,EPU 2.12.1.2782, TIA 5.0.0.2896, FluCam viewer 6.15.3.22415, Spectra Manager(Version 2.9.0.7), Spectra Manager (Version 2.15.01)

Data analysis

cryoSPARC v4.1
cryoSPARC live v4.1
ISOLDE 1.5
Coot 0.98
Phenix v1.20.1-4487)
ChimeraX 1.5
OriginPro 2022b
Dragonfly 2022_2
Relion 4
ctffind 4
WARP 1.1.0 beta
Amira 3D 2022
AreTomo 1.1.0
Morphometrics Toolkit v0.2
ImageJ/Fiji 2.14
PyHI 8a98c25
CTFfind 4

For manuscripts utilizing custom algorithms or software that are central to the research but not yet described in published literature, software must be made available to editors and reviewers. We strongly encourage code deposition in a community repository (e.g. GitHub). See the Nature Portfolio guidelines for submitting code & software for further information.

Data

Policy information about availability of data

All manuscripts must include a data availability statement. This statement should provide the following information, where applicable:

- Accession codes, unique identifiers, or web links for publicly available datasets
- A description of any restrictions on data availability
- For clinical datasets or third party data, please ensure that the statement adheres to our policy

The EMDB accession numbers for cryo-EM maps are EMD IDs:
18384, 18421, 18420, 18422, 18423, 18424, 18425, 18426, 18427, 18428, 18429, 18430, 18431, 18432, 18433, 18434, 18435, 18620, 19863, 19864, 19865, 19866, 19899, 19900, 19901, 19902, 19903, 19904
The PDB accession codes for Vipp1 models are PDB IDs:
8QFV, 8QHW, 8QHV, 8QHX, 8QHY, 8QHZ, 8QI0, 8QI1, 8QI2, 8QI3, 8QI4, 8QI5, 8QI6, 9EOM, 9EON, 9EOO, 9EOP
Available datasets accessed in this Manuscript are: PDB-ID: 7O3Y, 7O3X, 7O3W, 7O3Z

Research involving human participants, their data, or biological material

Policy information about studies with human participants or human data. See also policy information about sex, gender (identity/presentation), and sexual orientation and race, ethnicity and racism.

Reporting on sex and gender	N.A.
Reporting on race, ethnicity, or other socially relevant groupings	N.A.
Population characteristics	N.A.
Recruitment	N.A.
Ethics oversight	N.A.

Note that full information on the approval of the study protocol must also be provided in the manuscript.

Field-specific reporting

Please select the one below that is the best fit for your research. If you are not sure, read the appropriate sections before making your selection.

☒ Life sciences ☐ Behavioural & social sciences ☐ Ecological, evolutionary & environmental sciences

For a reference copy of the document with all sections, see nature.com/documents/nr-reporting-summary-flat.pdf

Life sciences study design

All studies must disclose on these points even when the disclosure is negative.

Sample size

Trp Fluorescence n=4, CDspectroscopy n=3, CryoEM SPA: Vipp1+EPL n=3217 micrographs, Vipp1 H1-6 n=5901 micrographs, Vipp1 H1-6 EPL

Sample size	n=2463 micrographs, Vipp1 (dL10Ala) EPL n=29548 micrographs, CryoET: 176 tilt series<br>Sample sizes were chosen based on instrument access time. The number of micrographs was sufficient to produce high resultion reconstructions of the samples.
Data exclusions	Micrographs of poor particle coverage and ice quality were discarded.
Replication	Due to the time-consuming nature of image acquisition and the limited access to this specialized microscope equipment, exact replicates were not performed.<br>Biochemical in vitro assay were repeated as described in the figure legends.
Randomization	Randomization is not applicable to the study because of the time-consuming nature of image acquisition and the limited access to this specialized microscope equipment (high-end Krios and Arctica microscopes).<br>Biochemical In vitro assays were not randomized, as this is not conventionally used in biochemical in vitro assys.
Blinding	Blinding experiments is not applicable to this study because of time-consuming nature of image acquisition and the limited access to this specialized microscope equipment (high-end Krios and Arctica microscopes).<br>Biochemical In vitro assays used objective quantification methods that are not susceptible to bias, so samples were not blinded.

Reporting for specific materials, systems and methods

We require information from authors about some types of materials, experimental systems and methods used in many studies. Here, indicate whether each material, system or method listed is relevant to your study. If you are not sure if a list item applies to your research, read the appropriate section before selecting a response.

Materials & experimental systems

n/a	Involved in the study
☒	☐ Antibodies
☒	☐ Eukaryotic cell lines
☒	☐ Palaeontology and archaeology
☒	☐ Animals and other organisms
☒	☐ Clinical data
☒	☐ Dual use research of concern
☒	☐ Plants

Methods

n/a	Involved in the study
☒	☐ ChIP-seq
☒	☐ Flow cytometry
☒	☐ MRI-based neuroimaging

Plants

Seed stocks	N.A.
Novel plant genotypes	N.A.
Authentication	N.A.
